# Delayed Release of Intracellular Microcystin Following Partial Oxidation of Cultured and Naturally Occurring Cyanobacteria

**DOI:** 10.3390/toxins12050335

**Published:** 2020-05-20

**Authors:** Katherine E. Greenstein, Arash Zamyadi, Caitlin M. Glover, Craig Adams, Erik Rosenfeldt, Eric C. Wert

**Affiliations:** 1 Southern Nevada Water Authority (SNWA), P.O. Box 99954, Las Vegas, NV 89193-9954, USA; katie.greenstein@gmail.com; 2 Water Research Australia (WaterRA), Adelaide, SA 5001, Australia; arash.zamyadi@waterra.com.au; 3 BGA Innovation Hub and Water Research Centre, School of Civil and Environmental Engineering, University of New South Wales (UNSW), Sydney, NSW 2052, Australia; 4 Department of Civil Engineering, McGill University, Montreal, QC H3A 0G4, Canada; caitlinmeara@gmail.com; 5 Department of Civil Engineering, Saint Louis University, St. Louis, MO 63103, USA; craig.adams@slu.edu; 6 Hazen and Sawyer, Raleigh, NC 27607, USA; erosenfeldt@hazenandsawyer.com

**Keywords:** cyanobacteria, oxidation, stagnation, microcystin, water treatment, quenching

## Abstract

Oxidation processes can provide an effective barrier to eliminate cyanotoxins by damaging cyanobacteria cell membranes, releasing intracellular cyanotoxins, and subsequently oxidizing these toxins (now in extracellular form) based on published reaction kinetics. In this work, cyanobacteria cells from two natural blooms (from the United States and Canada) and a laboratory-cultured *Microcystis aeruginosa* strain were treated with chlorine, monochloramine, chlorine dioxide, ozone, and potassium permanganate. The release of microcystin was measured immediately after oxidation (t ≤ 20 min), and following oxidant residual quenching (stagnation times = 96 or 168 h). Oxidant exposures (CT) were determined resulting in complete release of intracellular microcystin following chlorine (21 mg-min/L), chloramine (72 mg-min/L), chlorine dioxide (58 mg-min/L), ozone (4.1 mg-min/L), and permanganate (391 mg-min/L). Required oxidant exposures using indigenous cells were greater than lab-cultured *Microcystis*. Following partial oxidation of cells (oxidant exposures ≤ CT values cited above), additional intracellular microcystin and dissolved organic carbon (DOC) were released while the samples remained stagnant in the absence of an oxidant (>96 h after quenching). The delayed release of microcystin from partially oxidized cells has implications for drinking water treatment as these cells may be retained on a filter surface or in solids and continue to slowly release cyanotoxins and other metabolites into the finished water.

## 1. Introduction

Toxic cyanobacteria blooms are a public health risk when present in drinking water supplies. Many countries have issued health advisories for cyanotoxins, including microcystin, a hepatotoxic class of cyanotoxins with many different variants, or congeners [1,2,3]. In response to these cyanotoxin health advisories, drinking water utilities often develop cyanobacteria bloom (frequently termed “harmful algal bloom” (HAB)) management plans to prevent the persistence of cyanotoxins beyond treatment [4,5]. Furthermore, several guidance documents emerged with best practices for managing cyanobacteria blooms in water treatment [5,6,7,8,9]. Recommendations include switching water sources and removing cells through conventional treatment processes (i.e., dissolved air flotation, sedimentation).

Many utilities use pre-oxidation in drinking water treatment to achieve a multitude of objectives including invasive species control (e.g., quagga mussel), prevention of biofilm growth, taste and odor control, oxidation of iron or manganese, improved solids removal, and disinfection [10]. Current guidance also urges caution or recommends eliminating pre-oxidation processes to minimize the risk of cell lysis and the corresponding release of intracellular cyanotoxin before the removal of intact cells (e.g., through coagulation/flocculation/sedimentation and/or filtration). Despite this guidance, some utilities cannot eliminate pre-oxidation in order to comply with disinfection requirements (e.g., *Giardia* inactivation) [11]. Therefore, guidance for the application of pre-oxidants during a cyanobacteria bloom is warranted. While previous studies have generally assessed the complete oxidation of cyanobacteria cells and release of cyanotoxins, particularly with respect to microcystin [12,13,14,15,16,17,18,19], low oxidant exposures (concentration × time (CT)) resulting in incomplete cell oxidation continue to require further examination. Furthermore, delayed release has been observed up to 120 min after the chlorination of cells [17]. Additional research is needed to understand the risk of additional intracellular cyanotoxin release following partial cyanobacteria cell oxidation and oxidant quenching processes using different oxidants (ozone, chloramine, chlorine dioxide, potassium permanganate) in water treatment plants.

Here, microcystin release from both laboratory-cultured and naturally occurring cyanobacteria was assessed with exposure to drinking water pre-oxidation. By normalizing the oxidant dose to the dissolved organic carbon (DOC) concentration of each water, chlorine, monochloramine, chlorine dioxide, ozone, and potassium permanganate were compared in three cyanobacteria suspensions. The objectives of the research were to: (1) establish a normalized framework (i.e., oxidant dose: background DOC ratio, oxidant exposure over time) where the complete release of intracellular microcystin and DOC may be expected following a 20 min reaction time, and (2) examine the delayed release of intracellular microcystin following partial cell damage at low oxidant exposures with stagnation times ranging from 5 min to 7 days. This work improves the guidance regarding continued intracellular microcystin release from partially damaged cells that can impact downstream water treatment processes (i.e., filter surface, solids retention basins).

## 2. Results and Discussion

### 2.1. Oxidation (Time ≤ 20 min) Induced Degradation of Pigment and Release of DOC

**Figure 1:**
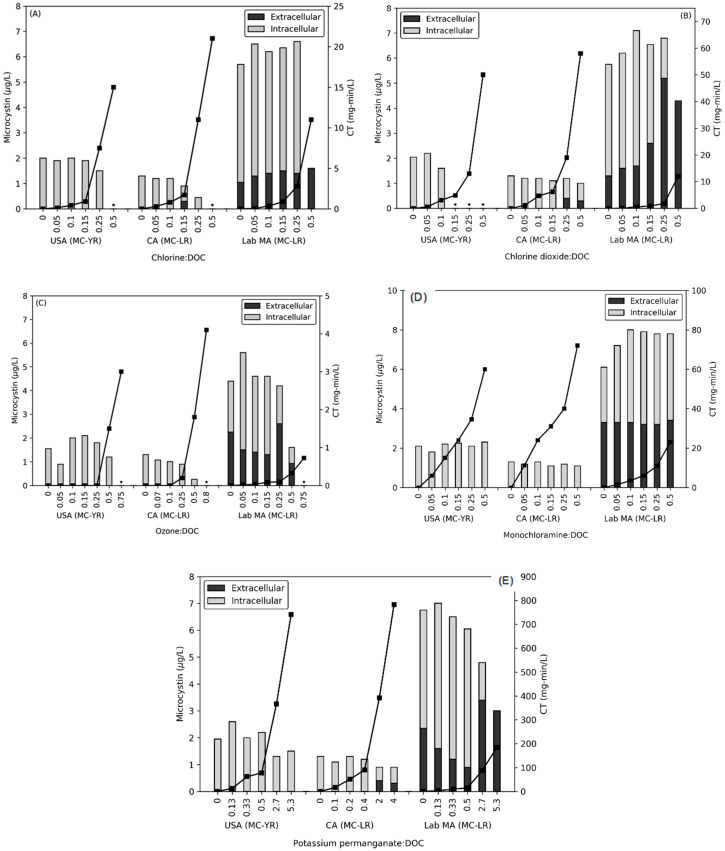
Impact of (**A**) chlorine, (**B**) chlorine dioxide, (**C**) ozone, (**D**) monochloramine, and (**E**) potassium permanganate on the release of intracellular MCs (MC-LR or MC-YR) from the USA bloom, the CA bloom, and the *M. aeruginosa* (lab MA) water (t ≤ 20 min). CT is shown on the secondary axis as black squares. * Concentrations were below the MRL for both extracellular and intracellular MC.

Across the three waters tested, all five oxidants were applied at either five or six oxidant: DOC dose ratios for contact times ≤ 20 min. The oxidant decay rates during this time period (Table S1) were calculated for each dose ratio, with decay curves shown in the SI (Figures S1–S10). Along with the decay rates, oxidant CT values were calculated for each oxidant using the trapezoid rule with two residual oxidant values. CT values are shown on the secondary axis of Figure 1A–E and in Table S2. Ozone CT values were nominally 0 mg-min/L after the USA bloom was dosed with 0.05–0.25 O_3_: DOC dose ratios and after the CA bloom was dosed with of 0.07–0.1 O_3_: DOC due to the rapid decay of ozone.

While cell viability was not directly monitored, chlorophyll-a (chl-*a*) (for the USA bloom and lab-cultured *M. aeruginosa*) and phycocyanin (PC) fluorescence (for the CA bloom) were used as proxies for cell damage. The chl-*a* or PC fluorescence were used to calculate cell damage rates with results shown in Table 1 and Table S3. In general, the cell damage rates followed the trend of ozone >> chlorine ≈ chlorine dioxide > potassium permanganate >> monochloramine. The USA and CA bloom waters had significantly lower decay rates for all oxidants as compared to the lab-cultured *M. aeruginosa*. This phenomenon is due to the resilience of natural (indigenous) cells with presence of multiple species and the interference from background organic matter, compared to lab-cultured species, which has been observed previously [16,20,21,22]. However, at the low pre-oxidation doses, ozonation (1700 M^−1^s^−1^) and chlorine dioxide (200 M^−1^s^−1^) rates for the lab-cultured *M. aeruginosa* were significantly lower than those rates observed in past work (1.1 × 10^5^ M^−1^s^−1^ and 4900 M^−1^s^−1^) [23]. The potassium permanganate rate (9.1 M^−1^s^−1^) was comparable, though lower, than the value generated (36 M^−1^s^−1^) for another unicellular species, *Pseudanabaena sp.*, likely due to differences in cell-specific reactivity as similar experimental conditions were applied [24]. These data illustrate that during low pre-oxidation doses or the first part of the cell-damage curve, the rate is significantly lower. A caveat in comparing the pigment results from the three waters is that the PC fluorescence (used for the CA bloom) likely captured both intra- and extra-cellular pigment, whereas the extracted chl-*a* (used for the USA bloom and lab-cultured water) included only intracellular pigment [25].

While pigment levels are a proxy for cell-viability, the release of DOC is an indicator of cell lysis. At the highest oxidant:DOC dose ratios for chlorine (0.5), monochloramine (0.5), chlorine dioxide (0.5), potassium permanganate (4/5.3), and ozone (0.75/0.80) DOC increased by less than 1.5 mg/L during the ≤20 min exposure period. In each of the three waters, ozone produced the greatest releases of 0.91 mg/L in the USA bloom, 1.20 mg/L in the CA bloom, and 0.30 mg/L in the lab-cultured *M. aeruginosa* water. After exposure to chlorine, monochloramine, chlorine dioxide, and potassium permanganate, DOC releases were consistent across the three waters at 0.30–0.65 mg/L for USA bloom, 0.1–0.2 mg/L for the CA bloom, and 0.2–0.26 mg/L for the lab-cultured *M. aeruginosa*. A potential consequence of the release of DOC during pre-oxidation is that these processes may result in the formation of disinfection byproducts during the initial oxidation period or later during secondary disinfection as has been demonstrated in previous work [17,21,26]. The presence of released DOC may also interfere with the degradation of extracellular MC as it consumes oxidant residual during the exposure period and is a factor contributing to the difficulty in modeling the degradation of MC as it moves from intracellular to extracellular [27].

### 2.2. Impact of Oxidants on Release of Microcystin (t ≤ 20 min)

To monitor the release of MC congeners from the USA bloom (MC-YR), CA bloom (MC-LR) and the lab-cultured *M. aeruginosa* (MC-LR), the total and extracellular MC were measured (Figure 1A–E). Prior to oxidation, the highest concentration of total MC was observed in the lab-cultured *M. aeruginosa* sample, 5.7 ± 0.86 μg/L MC-LR, whereas the bloom waters contained significantly less at 1.9 ± 0.22 μg/L MC-YR in the USA bloom, and 1.3 μg/L MC-LR in the CA bloom. The extracellular MC started at concentrations below the method reporting limit (MRL, i.e., <0.5 μg/L) in the bloom waters, but the lab culture contained approximately 30% extracellular MC-LR relative to the total.

Pre-oxidation exposures resulted in the release of intracellular MC from both natural bloom and lab-cultured cyanobacteria as summarized in Table 2 Two of the oxidants, monochloramine and potassium permanganate, saw limited release of intracellular MC. For all three waters, the highest dose ratio of monochloramine (CTs of 23–72 mg-min/L) resulted in a release less than 0.15 μg/L of extracellular MC and no significant total MC degradation. This corresponds with the low rate of monochloramine inactivation and reaction with cell membrane [15,28]. In past work, with lab-cultured cells the release of MC-LR by monochloramine required a CT value more than an order of magnitude greater (640 mg-min/L for 5 × 10^4^ cells/mL) [13].

Potassium permanganate did not affect the MC levels in the two bloom samples until the two highest doses of KMnO_4_: DOC (CTs of 88–782 mg-min/L). Both blooms then saw a minor decrease in the total MCs with the CA bloom releasing an average of 0.35 μg/L as extracellular MC-LR. The lab-cultured *M. aeruginosa* water released intracellular MC at the two highest dose ratios, with the highest dose containing 3 μg/L of extracellular MC-LR. Prior to the release of intracellular MC, extracellular MC-LR was oxidized by KMnO_4_ [29,30].

The impacts of chlorine, chlorine dioxide, and ozone on the total and extracellular MC in the lab-cultured and bloom-containing waters are illustrated in Figure 1A–C, respectively. During the application of ozone, no extracellular MC was detected in the two blooms and this was attributed to the rapid oxidation of MC by ozone (0.24–4.1 × 10^5^ M^−1^s^−1^) and hydroxyl radical (1.1 × 10^10^ M^−1^s^−1^) prior to measurement [31]. Intracellular MC-YR in the USA bloom was removed to below the MRL at 0.5 Cl_2_: DOC and 0.15 ClO_2_: DOC, with no extracellular MC detected. Although chlorine (33 M^−1^s^−1^) and chlorine dioxide (1 M^−1^s^−1^) both have significantly lower reaction rates than ozone, a similar result was observed [29,32]. The CA bloom released extracellular MC-LR at 0.15 Cl_2_: DOC and 0.25 ClO_2_: DOC. Subsequent treatments with chlorine degraded the total MC from the CA bloom, but chlorine dioxide allowed both intra- and extracellular MC to remain. In contrast, lab-cultured *M. aeruginosa* released intracellular MC at the lowest dose ratios of 0.05 Cl_2_: DOC or ClO_2_: DOC (CT = 0.047 mg-min/L for chlorine and 0.060 mg-min/L for chlorine dioxide). This trend continued for both oxidants until the highest doses at which point the total MC was entirely extracellular. Ozonation of the *M. aeruginosa* degraded the extracellular MC until 0.5 O_3_: DOC, at which point 1.3 μg/L was released.

To model the degradation of total MC (k_total_), Equation (1) with CT was applied [12,14,16] with results shown in Table 1 and Table S3. The USA bloom, CA bloom, and the lab-cultured *M. aeruginosa* had ozone k_total_ rates of 143 M^−1^s^−1^, 56 M^−1^s^−1^ and 2664 M^−1^s^−1^, respectively. The bloom water rates were lower than those observed in a previous study of mixed species from a bloom (400–450 M^−1^s^−1^), likely due to the consumption of oxidant by the background organic matter in this work [20]. Under chlorination, the USA bloom’s MC-YR decayed at a rate of 102 M^−1^s^−1^, which was greater than the CA bloom MC-LR rate at 82.9 M^−1^s^−1^. This behavior could be partly due to the reactivity difference between the congeners with MC-YR more rapidly degraded as compared to MC-LR [33,34]. The lab-cultured sample in this work had a rate of 136 M^−1^s^−1^, a value that was close to those generated in past work (range of 10–96 M^−1^s^−1^) [12,14]. Although the cell-damage rates were an order of magnitude lower than those observed in previous studies, pre-oxidation k_total_ decay rates were close to those from previous studies. This reflects that lower oxidant doses are required to release MC relative to the doses necessary to induce changes in chl-*a* and PC fluorescence or release DOC.

These results demonstrate that extracellular MC can be released from lab-cultured *M. aeruginosa* cells at low oxidant: DOC (0.05 Cl_2_: DOC, 0.05 ClO_2_: DOC, 0.25 O_3_: DOC, and 2.7 KMnO_4_: DOC). Although this result has not been previously reported for chlorine dioxide nor ozone, a recent study with chlorine saw similar behavior [17]. It is important to note that the natural bloom waters were substantially more resistant to oxidation as compared to cultured cells as a result of the presence of multiple species, cell-specific resilience, and the interference from background organic matter [16,20]. In the USA bloom, no intracellular MC was released; however, total MCs were completely removed at 0.5 Cl_2_: DOC, 0.15 ClO_2_: DOC, and 0.75 O_3_: DOC. In the CA bloom, extracellular MC was observed at 0.15 Cl_2_: DOC, 0.25 ClO_2_: DOC, 0.8 O_3_: DOC, and 2 KMnO_4_: DOC, but complete removal did not occur.

### 2.3. Impact of Stagnation (Time Max = 96 or 168 h) on Pigment and DOC

After the initial 20 min oxidant exposure time, the remaining oxidant was quenched, and samples were subsequently held for up to 96 or 168 h. During the stagnation period, the release of DOC and cell damage (as shown via pigments concentration) was then evaluated. The dose ratios applied for chlorine, monochloramine, chlorine dioxide, ozone, and potassium permanganate were 0.15, 0.15, 0.15, 0.15, and 0.4/0.5, respectively. These dose ratios were selected as they produced minimal or no MC release during the initial oxidant exposure period.

**Figure 2:**
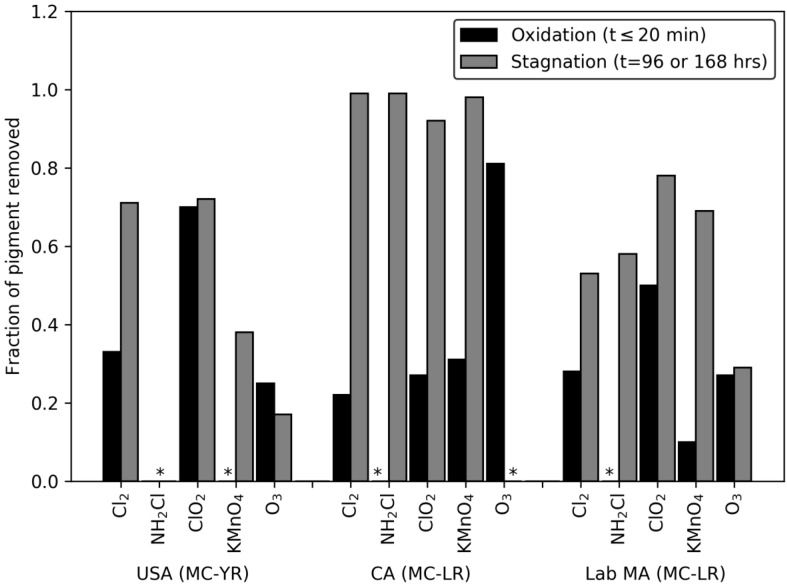
Fraction of pigment removed after 20 min exposure to oxidant: DOC ratios of 0.15. Cl_2_: DOC, ClO_2_: DOC, NH_2_Cl: DOC, and O_3_: DOC, and 0.5/0.4 KMnO_4_: DOC, and fraction of pigment removed after oxidation at the same level, quenching and stagnation for 96 or 168 h. Columns with * are those in which the pigment concentrations did not change or increased.

Interestingly, the reduction in pigment during the oxidant exposure time followed an expected degradation path and pseudo-first order reaction kinetics were applied to model the reduction and interpret the results. However, the model method does not fit the pigment decay rates analysis after quenching and stagnation time, i.e., the majority of R^2^ values are below 0.75. Therefore, the fraction of damaged cells after oxidation (time ≤ 20 min) were compared against the cells damaged during the stagnation period (Figure 2). All the ozone dose ratios produced a similar level of damage during the initial oxidation period and following stagnation. Following chlorine, potassium permanganate, and monochloramine treatment, the stagnation period more than doubled the fraction of cells classified as damaged relative to those affected during the oxidation period. In the CA bloom, after stagnation, the level of cell damage was the same for chlorine, monochloramine, chlorine dioxide, and potassium permanganate. The USA bloom and the lab-cultured *M. aeruginosa* saw that potassium permanganate, chlorine, and chlorine dioxide damaged a higher fraction of cells than ozone did after stagnation. This is in contrast with the general reactivity of these oxidants with cell membranes as well as their reactivity with organic matter functional groups [28,35]. It also does not agree with the pigment decay rates that were observed in this work after time ≤ 20 min contact. These results imply that oxidant-induced cell damage is strongly underestimated by the rates calculated using oxidant exposures that only measure cells immediately following oxidant exposure.

**Figure 3:**
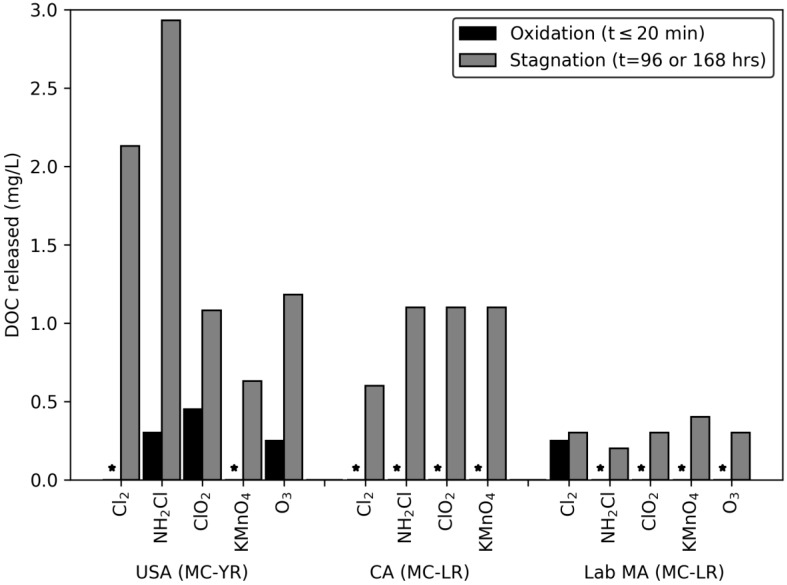
Release of DOC following exposure to oxidant: DOC ratios of 0.15 Cl_2_: DOC, ClO_2_: DOC, NH_2_Cl: DOC, and O_3_: DOC, and 0.5/0.4 KMnO_4_: DOC with 20 min contact time. Stagnation samples were collected 96 or 168 h after quenching. Columns with * represent releases below the MRL of 0.15 mg/L.

A similar trend was observed for the DOC released following stagnation (Figure 3). The initial exposure period produced limited releases of DOC above 0.15 mg/L, but after stagnation every oxidant produced a release of DOC. In addition, the changes in DOC concentration did not follow the trend of oxidant reactivity. This ranged from 0.63–2.93 mg/L for the USA bloom, 0.6–1.1 mg/L for the CA bloom, and 0.16–0.3 mg/L for the lab-cultured *M. aeruginosa*. As with the release of DOC during the initial oxidation period, the intracellular DOC could contribute to the formation of disinfection byproducts and interfere with the degradation of MC.

### 2.4. Impact of Stagnation (Time Max = 96 or 168 h) on Microcystin

While many studies have evaluated the impact of oxidation on the release of MC, limited work has been done to evaluate the impact of stagnation post-quenching. This is a particularly important process to understand for the use of pre-oxidation, as the immediate release might not occur (results shown above) leading to a misevaluation of the risk associated with a given oxidation dose. In this work, one dose ratio was selected, and the extracellular and total MC were tracked after quenching for up to 96 or 168 h.

**Figure 4:**
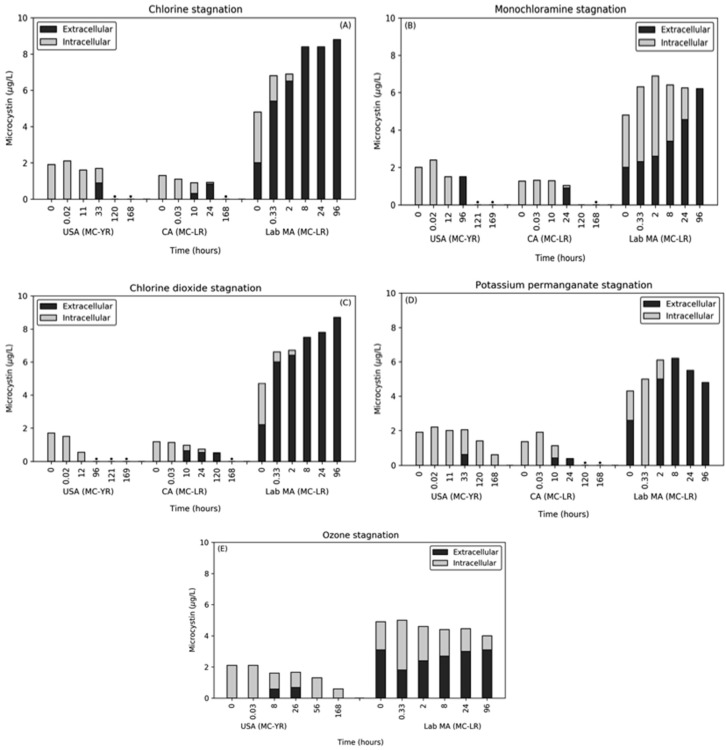
Effect of stagnation time on the presence of extracellular and intracellular MC after. oxidation and quenching with (**A**) chlorine, (**B**) monochloramine, (**C**) chlorine dioxide, (**D**) potassium permanganate, and (**E**) ozone. * Concentrations were below the MRL for both extracellular and intracellular MC.

Across all oxidants, the naturally occurring bloom samples did not immediately release MC during stagnation (Figure 4). For chlorine, monochloramine, ozone, and potassium permanganate, the release of MC-YR in the USA sample occurred at 33 h, 24 h, 8 h, and 33 h, respectively. Except for potassium permanganate and ozone where 0.61 and 0.68 μg/L of intracellular MC remained, the final time points were below the MRL for both intra- and extracellular MC-YR. For the CA bloom, extracellular MC-LR was observed after 10 h for chlorine, 24 h for monochloramine, 10 h for chlorine dioxide, and 10 h for potassium permanganate. Despite the limited release of MC during the initial oxidant exposure, the cells were damaged to the point where after additional time, they released MC. In both bloom waters, a decrease in total MC was observed over time, despite the conversion of this MC from intracellular to extracellular. This decrease was attributed to biodegradation, which will be further discussed below.

The lab-cultured *M. aeruginosa* was significantly more susceptible to the release of intracellular MC during stagnation with extracellular MC-LR detected after < 0.6 hrs. In contrast to the natural bloom samples, the final time point for the lab-cultured cells contained almost entirely extracellular MC. At 96 h, chlorine, monochloramine, chlorine dioxide, and potassium permanganate stagnation contained 8.8 μg/L, 6.2 μg/L, 8.7 μg/L, and 4.8 μg/L, respectively. Ozone was the only oxidant in which a small concentration of intracellular MC remained during stagnation with 3.1 μg/L extracellular and 0.9 μg/L intracellular. As with the bloom waters, the ozone stagnation resulted in a degradation of the total MC present. Stagnation after chlorine and chlorine dioxide exposure saw continued increases in the extracellular MC from first to last time point. The total MC in the potassium permanganate stagnation sample increased until 8 h at which point the MC was entirely extracellular and subsequent decay was observed.

The delayed release of MC from damaged or lysed cells highlights the need to monitor MC throughout water treatment and solids-handing, not only following the pre-oxidation process. It also provides another potential source of MC release during treatment, which had previously been attributed to the accumulation of cells during treatment, e.g., in filter beds, in the sludge bed of sedimentation tanks, and in sludge thickeners [36,37,38]. Using the release of extracellular MC-LR from the lab-cultured *M. aeruginosa*, the rate at which this process occurs was calculated using Equation (1) and ranged from 1.61 to 4.12 × 10^−6^ s^−1^ (Table 3). These rates were generated from the release following chlorine, chlorine dioxide, ozone, and monochloramine, and did not follow a specific trend with oxidant efficacy. For the bloom samples, total MC concentration decreased during stagnation and the continuous release of extracellular MC was not observed. In both the bloom samples, regardless of the oxidant, similar total MC decay rates were observed, indicating that the process was likely not related to the oxidant applied. For the USA bloom, the rates ranged from 1.79 to 2.57 × 10^−6^ s^−1^ after chlorination, ozonation, potassium permanganate, and monochloramine exposure. The CA bloom had slightly higher rates, but the spread between oxidants was also low, with potassium permanganate, chlorine dioxide, monochloramine, and chlorine at 2.85 to 4.08 × 10^−6^ s^−1^.

The degradation in these waters was attributed to biological activity and the rates were translated to half-lives for comparison with past work. The extracellular MC-LR half-life ranged from 3.12–4.48 days in the USA bloom and 1.97–2.81 days in the CA bloom; these half-lives were similar to those observed in surface waters (1.22–7.66 days for MC-LR) [39] and lake waters (5.4 days for both MC-LR and MC-YR) [40]. A lag phase was observed, but there was not a discernible relationship between the lag phase and the oxidant applied. The similarity in behavior between the two blooms indicated that both waters had the required biomass and the enzymes present for biodegradation, which is indicative of a previous exposure to cyanobacteria producing MC in these waters [39,40]. With the exception of ozonation, the lab-cultured *M. aeruginosa* in Colorado River Water (CRW) did not exhibit the biodegradation behavior observed in the bloom waters. Following the application of ozone, total MC-LR degraded at a rate of 7.44 × 10^−7^ s^−1^, which translated to a half-life of 10.8 days. This lower rate was indicative of the lower biomass present in the CRW and/or limited prior exposure to MC [40].

## 3. Conclusions

This study demonstrated differences between lab-cultured and natural-bloom cyanobacteria cell lysis rates, identified the CT values required for the complete intracellular release of microcystin, and identified the importance of stagnation following partial damage to cyanobacteria cells. Lab-cultured *M. aeruginosa* cells were found to be more susceptible to chemical oxidation than natural bloom cells. For lab-cultured *M. aeruginosa*, the level of extracellular MC increased after dose ratios of 0.05 Cl_2_: DOC, 0.05 ClO_2_: DOC, 0.25 O_3_: DOC, and 2 KMnO_4_: DOC. In contrast, the mixed species found in the two bloom waters required higher doses of 0.15 Cl_2_: DOC, 0.15 ClO_2_: DOC, 0.75/0.80 O_3_: DOC, and 2/2.7 KMnO_4_: DOC. Oxidant: DOC ratios and CT values that resulted in complete intracellular release were identified for each oxidant across the three water evaluated.

Following the oxidation phase and quenching, the effect of stagnation time was evaluated using partially damaged cells. Stagnation resulted in greater release of intracellular DOC and microcystin. At dose ratios of 0.15 for ozone, chlorine, chlorine dioxide, and monochloramine and a dose ratio of 0.4/0.53 for potassium permanganate, all three of the waters saw the release of extracellular MC. Increases in the extracellular fraction of MC started anywhere from 20 min after quenching for the lab-cultured sample up to 96 h after quenching for the USA bloom.

These data highlight the need to monitor the downstream time points for the potential release of extracellular toxin because partially damaged cells may be retained on filter surfaces or in sludge where continued MC release can threaten finished water quality and/or solids handling processes.

## 4. Materials and Methods

### 4.1. Cyanobacteria Culturing, Sampling, and Characterization

#### 4.1.1. Laboratory-Cultured *Microcystis aeruginosa* Cells Transferred into Colorado River Water (CRW)

A microcystin-LR (MC-LR) producing cyanobacteria strain, unicellular *Microcystis aeruginosa* (LB 2385, UTEX Austin Culture Collection, Austin, TX, USA), was cultured as described in prior work [41]. Briefly, cells were cultured in Bold3N media for ~30 days and subsequently rinsed with 10 mM phosphate buffer (pH 7.5) after three centrifugations, respectively. A cell stock solution was prepared by suspending cells in ~20 mL of the phosphate buffer. Cell concentration of the stock was determined via optical density at 730 nm (OD730), which was previously correlated with cell counts using a digital flow cytometer [23]. Subsequently, cells were spiked into Colorado River water (CRW), which had DOC of 2.5 mg/L, alkalinity of 138 mg/L as CaCO_3_, and pH of 8.0, to obtain 1.0 × 10^6^ cells/mL. The background DOC of CRW (2.5 mg/L) was used to determine the applied oxidant doses.

#### 4.1.2. United States (USA) Bloom: Grand Lake St. Marys

Grand Lake St. Marys in Celina, OH was sampled during October 2016, and the water was shipped in cubitainers on ice to Southern Nevada Water Authority (SNWA) for experiments. The USA bloom water had a DOC of 9.3 mg/L and pH of 7.9. The predominant cyanobacteria present was *Planktothrix agardhii/suspensa* (2.65 × 10^6^ cells/mL), followed by *Planktolyngbya spp.* (3.72 × 10^5^ cells/mL). The cyanobacteria were confirmed to produce microcystin-YR (MC-YR), which historically has been generated by *Planktothrix agardhii/suspensa* in the USA bloom [42]. Additional details and pictures of the bloom can be found in the Supporting Information (SI) Section S1.

#### 4.1.3. Canadian (CA) Bloom: Lake Champlain

*Anabaena spiroides* (1.58 × 10^5^ cells/mL), *Aphanothece clathrata brevis* (1.01 × 10^5^ cells/mL), and *Microcystis aeruginosa* (4.03 × 10^4^ cells/mL) complex naturally occurred on the Canadian side of Lake Champlain. Water was collected and transported to Polytechnique Montréal on ice by cubitainers. The Canadian bloom had a DOC of 6.1 mg/L and pH of 7.9.

### 4.2. Pre-Oxidation of Cyanobacteria Suspensions

#### 4.2.1. Varied Oxidant: DOC Ratios

Cyanobacteria suspensions were placed in 1 L amber glass bottles for exposure to oxidants. Chlorine (Cl_2_), chlorine dioxide (ClO_2_), monochloramine (NH_2_Cl), ozone (O_3_), and potassium permanganate (KMnO_4_) were assessed as pre-oxidants. Chlorine was obtained as a ~5% sodium hypochlorite (NaOCl) solution (Fisher Scientific). Chlorine dioxide was obtained as a ~3000 mg/L solution (CDG Environmental). KMnO_4_ was obtained as ~900 mg/L solution (Ricca). Monochloramine was prepared using ammonium chloride, sodium hydroxide (NaOH), and NaOCl as described in [43]. Oxidant stock concentrations were measured to ensure accurate dosing.

Chlorine, chlorine dioxide, monochloramine, and ozone were spiked into suspensions with oxidant: DOC mass ratios of 0.05, 0.10, 0.15, 0.25, and 0.5. Ozone was also assessed at O_3_: DOC of 0.75. The DOC for ratio calculations was based on the background DOC of the source water. Therefore, any released DOC following cyanobacteria cell lysis was not factored into these ratios. Potassium permanganate was spiked into suspensions with ratios of 0.1, 0.25, 0.4, 2, and 4 for the CA bloom and ratios of 0.13, 0.33, 0.5, 2.7, and 5.3 for the USA bloom and lab-cultured *M. aeruginosa*. Oxidants were allowed to react for a maximum of 20 min at room temperature (20 °C). Aliquots (10 mL) were taken from reactors throughout experiments for residual measurements; volume taken did not exceed 10% of total reactor volume. When no residual remained or 20 min had elapsed, sodium thiosulfate (Na_2_S_2_O_3_) was added to reactors (targeting 100 mg/L in the reactor to ensure excess) to quench residual and/or to give all reactors the same treatment. Samples were then immediately taken for analysis of total and extracellular microcystins (MC), chl-*a* or phycocyanin (PC), and DOC. The middle oxidant: DOC ratio for each oxidant (except for ozone, which had an additional ratio assessed) had a duplicate reactor to assess reproducibility. Control reactors without chemical addition and with Na_2_S_2_O_3_ addition were sampled as well; Na_2_S_2_O_3_ did not have a measurable effect on MC, pigments, nor DOC.

#### 4.2.2. Varied Stagnation Times

For select oxidants: DOC ratios (0.15 for Cl_2_, ClO_2_, NH_2_Cl, and O_3_; 0.4 for KMnO_4_), discrete reactors with 500 mL of cell suspensions in 500 mL amber glass bottles were spiked with oxidants for sampling at different time points. Reactors were quenched with Na_2_S_2_O_3_ at 20 min (or earlier when sampled for time points before 20 min). Sample times ranged from within 1 min of oxidant addition to either 65 or 168 h to look at the delayed release of MCs following partial pre-oxidation of cells. Samples were collected for total and extracellular MC, chl-*a* or PC, and DOC. Control reactors (no oxidant exposure; with and without Na_2_S_2_O_3_) were also sampled at each time point.

### 4.3. Sample Analyses

#### 4.3.1. Cyanobacteria Bloom Characterization and Water Quality Parameters

Optical density at 730 nm (OD730)—used for estimating cell density of laboratory-cultured *Microcystis aeruginosa* stock and suspensions—was measured using an ultraviolet-visible light spectrophotometer (Hach DR 5000). A correlation between OD730 and cell counts was established using a digital flow cytometer (FlowCAM, Fluid Imaging Technologies, Yarmouth, ME, USA) as described in previous work [23]. USA bloom samples—one as collected and one preserved with 1% Lugol’s iodine—were shipped overnight to BSA Environmental Services (Beachwood, OH, USA) for cyanobacteria imaging, identification, and enumeration. CA bloom samples were also preserved with 1% Lugol’s iodine for identification and enumeration at Polytechnique Montréal.

DOC was measured using Standard Method (SM) 5310 B and pH by SM 4500-H+ B. Chl-*a* was analyzed via SM 10,200 H (APHA, 2012). PC was measured with the Total Algae sensor on a YSI EXO2 Multiparameter Sonde (YSI, Yellow Springs, OH, USA). The sensor was blanked with deionized water and samples were measured by relative fluorescence units (RFU) as described in past work by [44].

#### 4.3.2. Oxidant Residuals

Free and total chlorine residuals were measured by SM 4500-Cl G. Ozone residuals were measured using indigo trisulfonate via SM 4500-O3 [45]. Chlorine dioxide residuals were measured by 4500-ClO2 D. Permanganate residuals were measured by SM 4500-Cl G, with 1 mg/L free chlorine measurement equivalent to 0.891 mg/L KMnO_4_ [45]. Permanganate samples were first filtered with 0.22 μm pore size polyvinylidene fluoride filters (Millex) to remove particulate manganese oxides which can interfere with residual measurement.

#### 4.3.3. Microcystin

For extracellular MC, samples were immediately filtered using 0.45 μm pore size glass microfiber syringe filters (Whatman). To lyse all cells and quantify total MC, 10 mL samples were first frozen at −20 °C, thawed at 25 °C, and sonicated for 5 min prior to filtration. Samples were sonicated with a probe sonicator (Q500, QSonica, Newtown, CT, USA) outfitted with a ¼ inch microtip operated at 20 kHz with 200 μm amplitude/tip displacement requiring ~30 W power. Samples were kept in an ice bath to prevent overheating of the sample and probe. The sonication was pulsed, with 5 s on and 1 s off, until total sonication time reached 5 min. Freeze/thaw, sonication, and filtration of a control (20 μg/L MC-LR spiked into CRW) did not cause degradation of MC-LR.

MC concentrations were measured with liquid chromatography tandem mass spectrometry (LC-MS/MS) and enzyme-linked immunosorbent assay (ELISA). Samples were analyzed using EPA Method 546 for total MC via ELISA. LC-MS/MS samples were analyzed for eight microcystin congeners (MC-LA, MC-LF, MC-LR, MC-LW, MC-LY, MC-RR, MC-WR, and MC-YR) via liquid chromatography-tandem mass spectrometry (LC-MS/MS) as described in previous work [13] with method reporting limits (MRLs) for each congener at 0.5 μg/L.

#### 4.3.4. Calculation of Oxidant Decay, MC Decay or Release Rates, and Cell Damage Rates

To analyze the data generated in this work, four different rate constants were calculated using Equation (1).

*C* = *C*_0_ × *e*^−*kR*^ (1)

In Equation (1), *C* was the concentration [total or extracellular MC (μg/L), and chl-*a* (μg/L) or PC (RFU)] at a given time, *C*_0_ was the starting concentration [total or extracellular MC (μg/L), and chl-*a* (μg/L) or PC (RFU) prior to oxidant exposure], and k was the oxidant decay rate [s^−1^ or M^−1^s^−1^]. Two of the rates calculated employed time as R (k_biodegradation_ and k_release_) and two used CT (k_total_ for total MC and k_damage_ to model cell damage) as R.

## Figures and Tables

**Table 1:** Cell damage (k_damage_) and total MC decay (k_total_) after oxidation (t ≤ 20 min) for the USA and CA blooms. Rates with R^2^ below 0.75 were excluded (e.g., monochloramine). * PC fluorescence was measured instead of extracted chl-*a*.

Oxidant	Water	k_damage_ (M^−1^s^−1^) (R^2^)	k_total_ (M^−1^s^−1^) (R^2^)
Cl_2_	USA	25 (0.96)	74.6 (0.91)
	CA	133 * (0.98)	82.9 (0.96)
ClO_2_	USA	64 (0.76)	-
	CA	20.9 * (0.84)	11.5 (0.82)
KMnO_4_	USA	-	-
	CA	2.69 * (0.84)	1.22 (0.73)
Ozone	USA	273 (0.91)	143 (0.99)
	CA	245 * (0.75)	56 (0.75)

**Table 2:** CT, oxidant: DOC ratio and stagnation time resulting in the release of intracellular MCs. * Select natural blooms did not release detectable concentrations of microcystins at this point.

	Cl_2_	NH_2_Cl	ClO_2_	O_3_	KMnO_4_
Oxidant:DOC ratio (t ≤ 20 min)	0.5	No release	0.5 *	0.75/0.80	>2 *
CT_lab_ (mg-min/L)	11	23	12	0.72	117
CT_USA_ (mg-min/L)	15	60	50	3.0	486
CT_CA_ (mg-min/L)	21	72	58	4.1	391
Stagnation time *	≥2 h	≥8 h	≥20 min	≥8 h	≥2 h

**Table 3:** Total MC biodegradation during stagnation (k_biodegradation_) and the release rate for extracellular MC after stagnation (k_release_) for the *M. aeruginosa* (lab MA), USA and CA blooms. Decay rates with R^2^ values below 0.75 were not included. * Differentiates *k_release_ from **k_biodegradation_**.

Oxidant	Water	Stagnation (Time Max = 96 or 168 h)	
		k_biodegradation_ (R^2^)/*k_release_ (s^−1^) (R^2^)	Half-Life (Days)
Chlorine	USA	2.57 × 10^−6^ (0.93)	3.12
	CA	3.37 × 10^−6^ (0.99)	2.38
	Lab MA	*1.61 × 10^−6^ (0.67)	-
Mono-chloramine	USA	2.44 × 10^−6^ (0.88)	3.29
	CA	4.08 × 10^−6^ (0.95)	1.97
	Lab MA	*4.12 × 10^−6^ (0.79)	-
Chlorine dioxide	USA	-	-
	CA	2.85 × 10^−6^ (0.90)	2.81
	Lab MA	*2.79 × 10^−6^ (0.83)	-
KMnO_4_	USA	1.79 × 10^−6^ (0.83)	4.48
	CA	3.93 × 10^−6^ (0.81)	2.04
	Lab MA	-	-
Ozone	USA	1.90 × 10^−6^ (0.90)	4.22
	Lab MA	7.44 × 10^−7^ (0.76)/*2.50 × 10^−6^ (0.84)	10.8

## Supplementary Materials

The following are available online at https://www.mdpi.com/2072-6651/12/5/335/s1, Figures S1–S5: Oxidant decay in the USA bloom for the five different oxidant: DOC ratios applied in this work, Figures S6–S10: Oxidant decay in the lab-cultured *M. aeruginosa* in CRW for the five different oxidant: DOC ratios applied in this work, Figure S11: Photos of USA bloom cyanobacteria. Table S1: Oxidant decay rates for lab-cultured *M. aeruginosa* (lab MA) and USA bloom. Table S2: Oxidant exposure generated from the oxidant decay rates for lab-cultured *M. aeruginosa* (lab MA), USA and CA blooms. Table S3: Cell damage (k_damage_) and total MC decay (k_total_) decay after oxidation (t < 20 min) in the lab-cultured *M. aeruginosa*.
