# Supplementary material for: Delayed Release of Intracellular Microcystin Following Partial Oxidation of Cultured and Naturally Occurring Cyanobacteria
Source: Toxins (Basel). 2020 May 20;12(5):335. doi: 10.3390/toxins12050335 (PMC7291037; doi:10.3390/toxins12050335)
Supplement: Supplementary file 1 [file toxins-12-00335-s001.pdf]

Supplementary Materials: Delayed Release of Intracellular Microcystin Following Partial Oxidation of Cultured and Naturally Occurring Cyanobacteria

**Katherine E. Greenstein, Arash Zamyadi, Caitlin M. Glover, Craig Adams ,
Erik Rosenfeldt and Eric C. Wert**

S1. First order oxidant decay curves

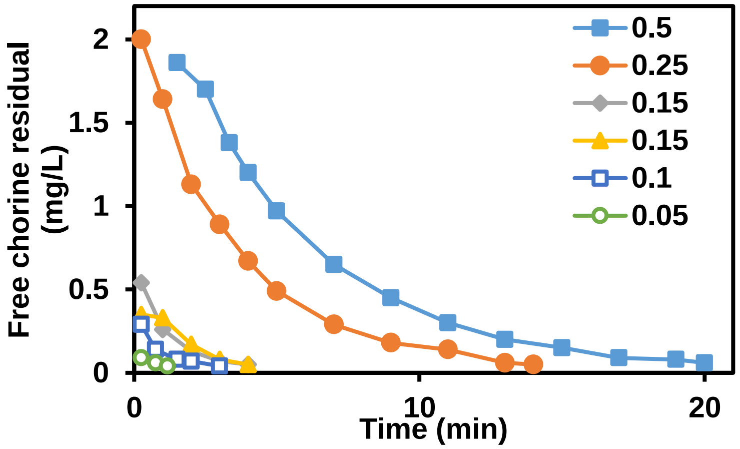

Figure S1. Chlorine residuals for five Cl_2:DOC ratios in the USA bloom.

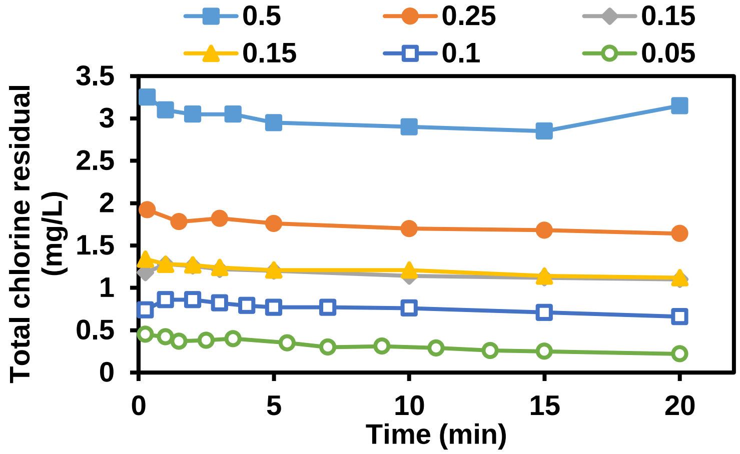

Figure S2. Total chlorine residuals for five different NH_2Cl:DOC ratios in the USA bloom.

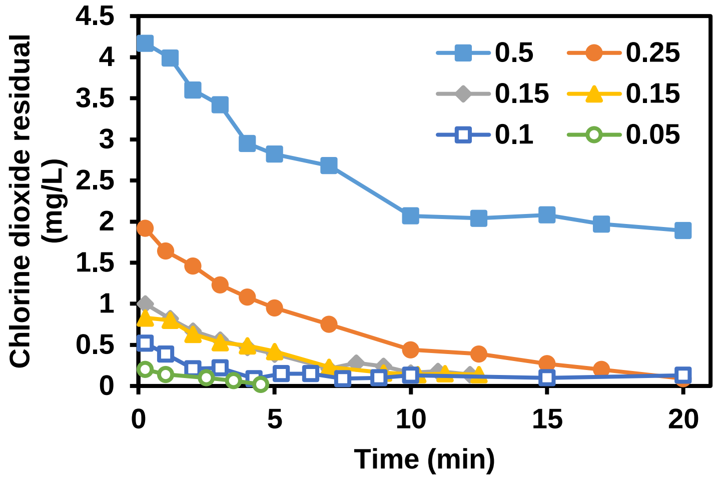

Figure S3. Chlorine dioxide residuals for five different ClO_2:DOC ratios in the USA bloom.

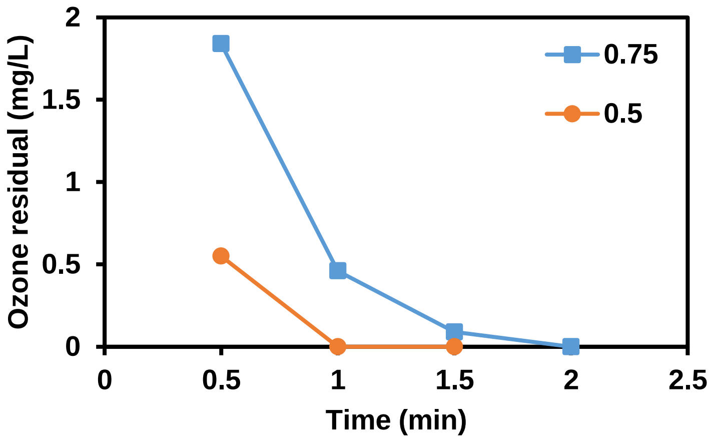

Figure S4. Ozone residuals at the two highest O_3:DOC ratios in the USA bloom. No residuals were detected for the other three O3:DOC dose ratios.

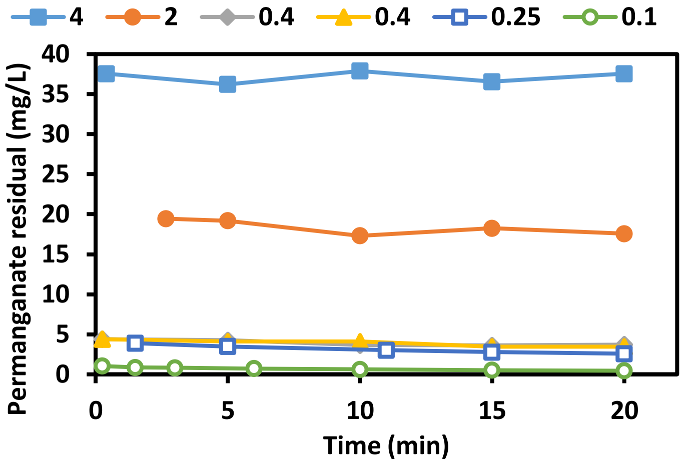

Figure S5. Potassium permanganate residuals for five $KMnO_4$:DOC ratios in the USA bloom.

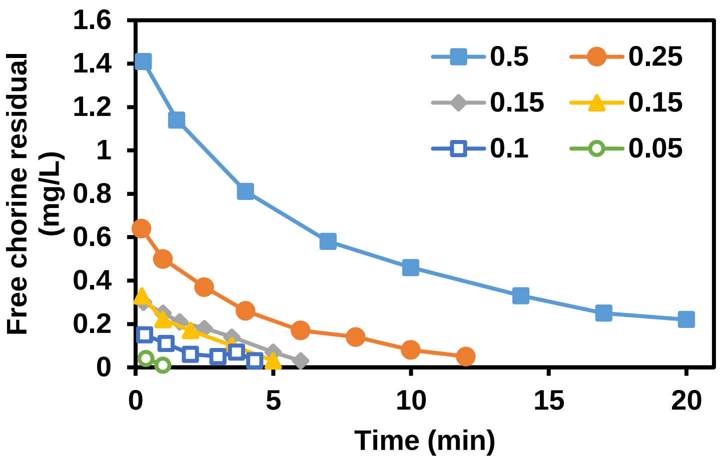

Figure S6. Chlorine residuals for five Cl_2:DOC ratios in the lab cultured *M. aeruginosa*.

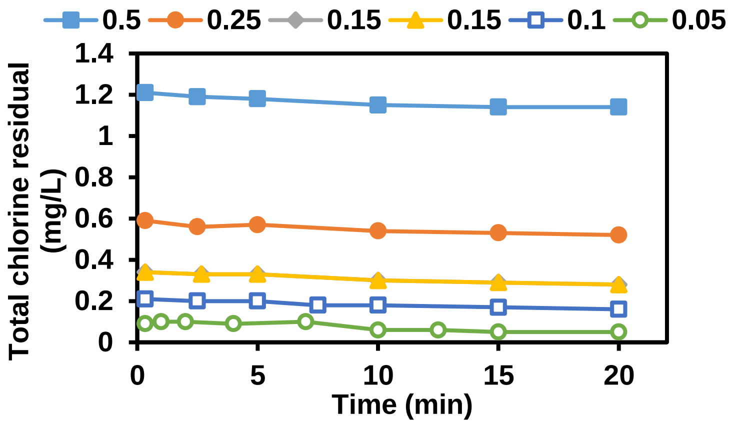

Figure S7. Total chlorine residuals for five NH_2Cl:DOC ratios in the lab cultured *M. aeruginosa*.

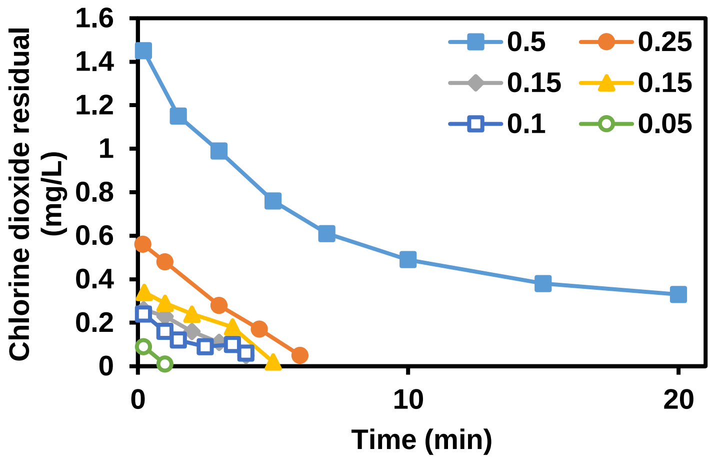

Figure S8. Chlorine dioxide residuals for five ClO_2:DOC ratios in the lab cultured *M. aeruginosa*.

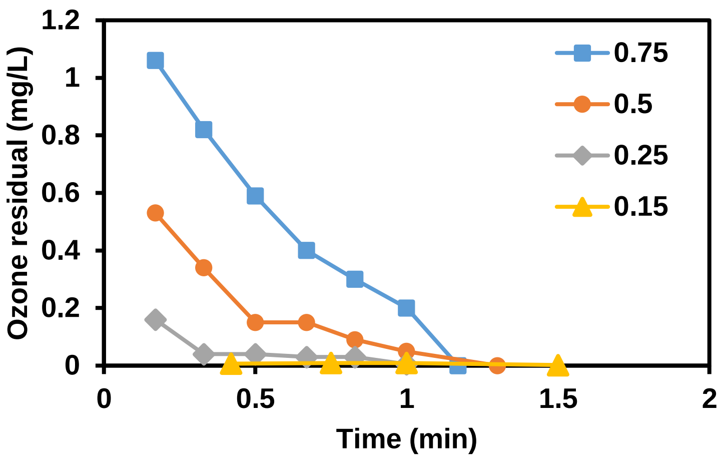

Figure S9. Ozone residuals at the four highest O_3:DOC ratios in the lab cultured *M. aeruginosa*. No residuals were detected for the lowest O_3:DOC dose ratio.

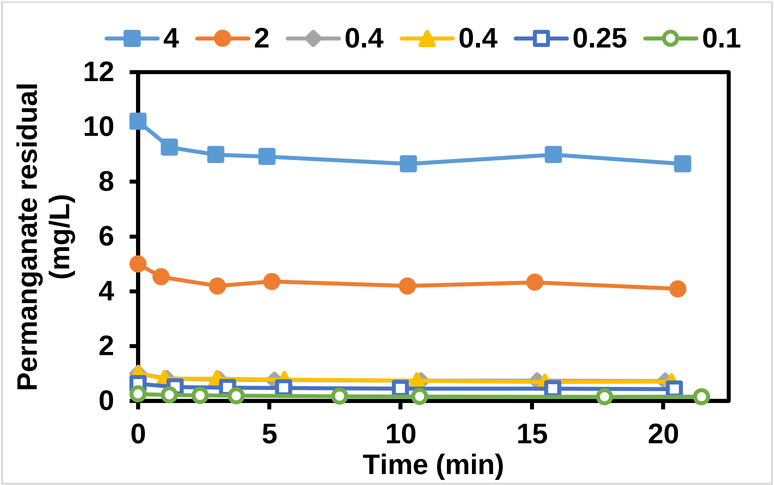

Figure S10. Potassium permanganate residuals for five $KMnO_4$:DOC ratios in the lab cultured *M. aeruginosa*.

Table S1. Oxidant decay rates for lab cultured *M. aeruginosa* (lab MA) and USA bloom. Rates with * were calculated from residuals < 1 minute. Decay curves with $R^2 < 0.75$ were not included and are marked with (x). In addition to the ozonation rate, the immediate ozone demand (%), and the ozone half-life (min) were calculated.

**Chlorine, monochloramine, or chlorine dioxide:DOC**		**k (s^{-1}) with R^2 in parentheses**				
		**0.5**	**0.25**	**0.15**	**0.1**	**0.05**
**Chlorine**	**USA**	0.0031 (0.99)	0.0043 (0.99)	0.0099±0.001 (0.99)	0.0090 (0.99)	0.030* (0.78)
	**Lab MA**	0.0015 (0.99)	0.0034 (0.99)	0.0074±0.0011 (0.94)	0.0055 (0.80)	0.042* (0.99)
**Mono-chloramine**	**USA**	9.70×10^{-5} (0.91)	8.50×10^{-5} (0.91)	$0.00012 \pm 1.1 \times 10^{-5}$ (0.93)	0.00022 (0.86)	0.00058 (0.95)
	**Lab MA**	4.37×10^{-5} (0.87)	8.31×10^{-5} (0.90)	0.000171 (0.95)	0.00021 (0.93)	0.00073 (0.86)
**Chlorine dioxide**	**USA**	0.00065 (0.88)	0.0024 (0.99)	0.0027±0.00011 (0.95)	0.0013 (0.47)	0.0088 (0.85)
	**Lab MA**	0.0011 (0.94)	0.0072 (0.92)	0.0094±0.0016 (0.88)	0.0042 (0.80)	0.044* (0.99)
**$KMnO_4$:DOC**		**5.3**	**2.7**	**0.5**	**0.33**	**0.13**
**$KMnO_4$**	**USA**	x	x	0.00023 (0.80)	0.00037 (0.99)	0.00060 (0.99)
	**Lab MA**	x	x	0.00097±0.0015 (0.97)	0.0011 (0.97)	0.015* (0.93)
**Ozone:DOC**		**0.75**	**0.5**	**0.25**		
**Ozone**	**USA**	0.048* (0.99); 38%; 0.24 min	x	x		
	**Lab MA**	0.036* (0.99); 31%; 0.32 min	0.050* (0.97); 40%; 0.23 min	0.065* (0.18); 30%; 0.26 min		

Table S2. Oxidant exposure generated from the oxidant decay rates for lab cultured *M. aeruginosa* (lab MA), USA, and CA blooms.

Chlorine, mono-chloramine, or chlorine dioxide:DOC		CT (mg-min L^{-1})				
		**0.5**	**0.25**	**0.15**	**0.1**	**0.05**
**Chlorine**	**USA**	15	7.5	0.91 ± 0.01	0.44	0.13
	**CA**	21	11	1.7	0.8	0.3
	**Lab MA**	11	2.8	0.88 ± 0.13	0.37	0.047
**Mono-chloramine**	**USA**	60	34.6	23.6 ± 0.46	15	6.1
	**CA**	72	40	31	24	11.3
	**Lab MA**	23	11	6.1	3.6	1.5
**Chlorine dioxide**	**USA**	50	13	4.85 ± 0.15	3.1	0.52
	**CA**	58	19	6.2	4.7	1.1
	**Lab MA**	12	1.8	0.9 ± 0.27	0.52	0.06
**$KMnO_4$:DOC**		**5.3**	**2.7**	**0.5**	**0.33**	**0.13**
**$KMnO_4$**	**USA**	741	366	77.8 ± 0.1	62.1	13
	**Lab MA**	184	88	15 ± 0.03	9.5	3.1
**$KMnO_4$:DOC**		**4**	**2**	**0.4**	**0.2**	**0.1**
**$KMnO_4$**	**CA**	782	391	90.4	51	17
**Ozone:DOC**		**0.75**	**0.5**	**0.25**	**0.15**	**0.1/0.05**
**Ozone**	**USA**	3.0	1.5	-	-	-
	**CA**	4.1	1.8	0.2	-	-
	**Lab MA**	0.72	0.33	0.10	0.089	0.034/ 0.018

Section S2. Impact of oxidation on pigments and microcystins

Table S3. Cell damage (k_{damage}) and total MC decay (k_{total}) decay after oxidation (t ≤ 20 min) in the lab-cultured *M. aeruginosa*.

Oxidant	k_{damage} ($M^{-1}s^{-1}$) (R^2)	Previous work ($M^{-1}s^{-1}$)	k_{total} ($M^{-1}s^{-1}$) (R^2)	Previous work ($M^{-1}s^{-1}$)
Cl_2	42 (0.62)	670 (Daly et al., 2007); 790 – 1100 (Lin et al., 2009); 756 – 1030 (Zamyadi et al., 2013); 2900 (Wert et al., 2013)	136 (0.94)	10 – 96 (Daly et al., 2007); 33 – 89 (Zamyadi et al., 2013)
ClO_2	200 (0.86)	4900 (Wert et al., 2013)	68 (0.88)	-
$KMnO_4$	9.1 (0.90)	36 (Li et al., 2018)	11 (0.95)	-
O_3	1700 (0.96)	1.1×10^5 (Wert et al., 2013)	2664 (0.98)	-

Section S3. USA bloom water details

In addition to the microcystin-YR (MC-YR) detected in the USA bloom, unidentified MC congeners or compounds with the -ADDA (3-amino-9-methoxy-2,6,8-trimethyl-10-phenyldeca;4,6-dienoic acid) group were detected using the Abraxis Microcystins-ADDA ELISA immunoassay. However, no match was found between commercially available standards (MC-LA, MC-LF, MC-LR, MC-LW, MC-LY, MC-RR, MC-WR, and MC-YR) and the LC-MS/MS results found in the USA bloom sample.

(**a**)

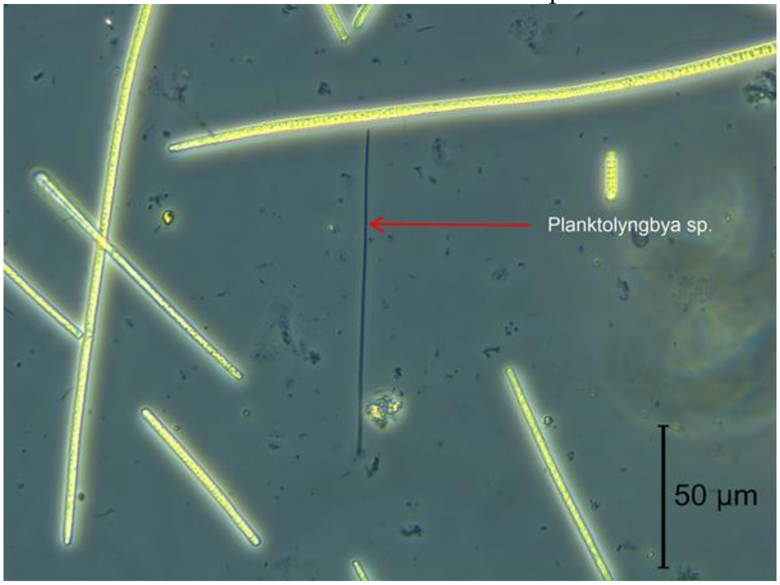

(**b**)

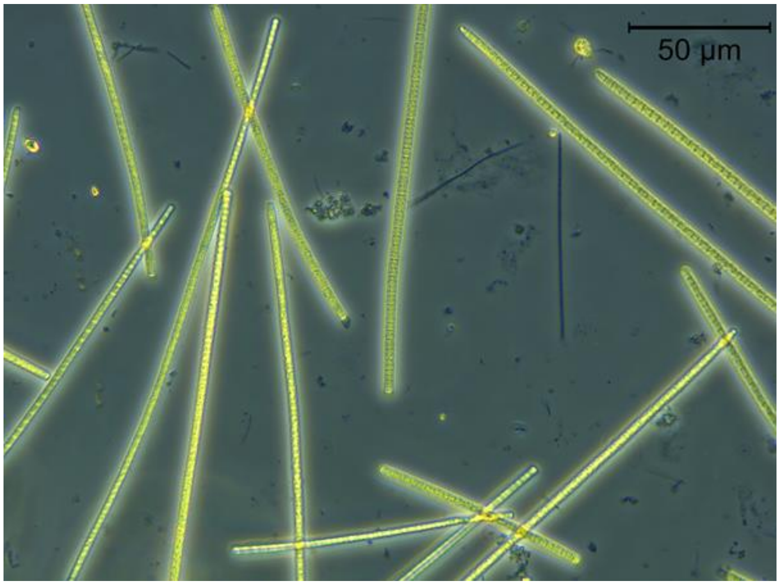

Figure S11. Photos of USA bloom cyanobacteria. (a) *Planktolyngbya spp.* at a 400x zoom (b) *Planktothrix agardhii/suspensa* with 400x zoom.
